# Robotic-assisted surgery for prostatectomy – does the diffusion of robotic systems contribute to treatment centralization and influence patients’ hospital choice?

**DOI:** 10.1186/s13561-023-00444-9

**Published:** 2023-05-10

**Authors:** David Kuklinski, Justus Vogel, Cornelia Henschke, Christoph Pross, Alexander Geissler

**Affiliations:** 1 Chair for Healthcare Management, School of Medicine, University of St. Gallen, St. Jakob-Strasse 21, 9000 St. Gallen, Switzerland; 2 Department of Health Care Management, Berlin University of Technology, Berlin Centre of Health Economics Research, Strasse Des 17. Juni 135, 10623 Berlin, Germany; 3 Department of Health Care Management, Berlin University of Technology, Strasse Des 17. Juni 135, 10623 Berlin, Germany

**Keywords:** Robotic-assisted surgery, Radical prostatectomy, Provider choice, Quality of care, Treatment centralization

## Abstract

**Background:**

Between 2008 and 2018, the share of robotic-assisted surgeries (RAS) for radical prostatectomies (RPEs) has increased from 3 to 46% in Germany. Firstly, we investigate if this diffusion of RAS has contributed to RPE treatment centralization. Secondly, we analyze if a hospital’s use of an RAS system influenced patients’ hospital choice.

**Methods:**

To analyze RPE treatment centralization, we use (bi-) annual hospital data from 2006 to 2018 for all German hospitals in a panel-data fixed effect model. For investigating RAS systems’ influence on patients’ hospital choice, we use patient level data of 4614 RPE patients treated in 2015. Employing a random utility choice model, we estimate the influence of RAS as well as specialization and quality on patients’ marginal utilities and their according willingness to travel.

**Results:**

Despite a slight decrease in RPEs between 2006 and 2018, hospitals that invested in an RAS system could increase their case volumes significantly (+ 82% compared to hospitals that did not invest) contributing to treatment centralization. Moreover, patients are willing to travel longer for hospitals offering RAS (+ 22% than average travel time) and for specialization (+ 13% for certified prostate cancer treatment centers, + 9% for higher procedure volume). The influence of outcome quality and service quality on patients’ hospital choice is insignificant or negligible.

**Conclusions:**

In conclusion, centralization is partly driven by (very) high-volume hospitals’ investment in RAS systems and patient preferences. While outcome quality might improve due to centralization and according specialization, evidence for a direct positive influence of RAS on RPE outcomes still is ambiguous. Patients have been voting with their feet, but research yet has to catch up.

## Background

Over the last decade, there has been a considerable surge in robotic-assisted surgeries (RAS) across the globe [1, 2]. Although the first RAS was already performed in 1985 by Kwoh et al. [3], RAS have taken off only with the latest technologic advancements – in particular with the authorization and market entry of the DaVinci Surgical System in 2000 [4]. In Germany, the number of RAS has increased by more than tenfold from 2,125 in 2008 to 23,724 in 2018, with strong further growth expected [5] (see Figure A1 in the Appendix). Globally, treatment areas most strongly affected by RAS systems are gynecology and urology (e.g., 75% of all RAS in the US in 2014 [6]) while in Germany, most RAS systems are still used for procedures on the male reproductive system. Specifically, by 2018, 46% of all radical prostatectomies (RPE), the main surgical treatment for localized prostate cancer, were robotic-assisted (up from 3% in 2008), covering roughly 50% of all RAS in 2018.

The trend towards an increased use of RAS systems in RPE can have a considerable impact on the hospital landscape performing urologic procedures. For example, Chang et al. [4] report a shift in the US towards fewer hospitals with higher case volumes for prostatectomies due to the surge of RAS. In Germany, contrary to the national trend of fewer prostatectomies between 2006 and 2013, hospitals with robotic systems were able to increase their annual case numbers [7]. Several factors on hospital and patient level are present that potentially contribute to this development: From the hospital perspective, clinicians might put pressure on hospital management to work with the most innovative technologies [8]. Besides a spill-over effect of physicians demanding to work with those technologies, RAS can considerably reduce physicians’ physical exhaustion and back pain, especially for longer surgeries [9]. The consequent high investment and maintenance costs of robotic systems then require hospitals to expand case volumes to economically break-even [10, 11]. Furthermore, there might be effects based on patient demand. Patients might be deliberately choosing hospitals with the latest surgical technologies. For instance, Boys et al. [12] show that despite a general low level of patient knowledge on what RAS  is, a majority of patients associates the existence of an RAS system in hospitals with better quality of care. This perception is likely driven by potential favorable scientific evidence and/or by effective marketing [13].

Consequently, we address the following research questions for the German hospital sector in our study:

1. How has the introduction of RAS systems affected treatment centralization and hospitals’ RPE procedure volumes?
2. Does the use of an RAS system influence patients’ hospital choice and therefore contribute to the changing hospital landscape for urologic procedures?

We then discuss our results in the light of first the scientific evidence for outcome quality improvements of RAS compared to more traditional surgical methods for prostatectomies, second the incentives for hospitals using robotic systems and robotic system producers to promote RAS, and third the effect robotic systems can have on workplace attractiveness for clinicians (physician health, recruitment, and retention).

For analyses of RPE treatment, we use (bi-) annual hospital data from 2006 to 2018 in a panel-data fixed effect model (see Methods). To determine the influence of RAS systems on patients’ hospital choice, we employ patient-level data from 2015 in a random utility choice model estimating the marginal utility a reference patient receives from the presence of an RAS system and we calculate patients’ willingness to travel for a hospital performing RAS (see Methods). The Results section reports the results. The Discussion section discusses implications and concludes.

## Methods

### Data

**Fig. 1:**
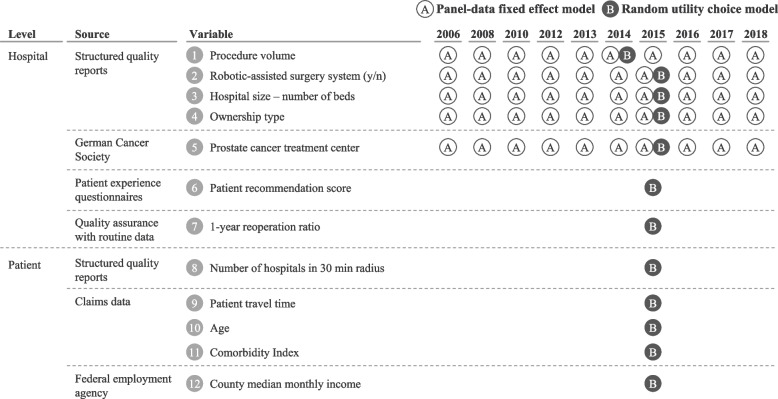
Variables and data sources for the panel-data fixed effect and the random utility choice model in one line *Notes:* Each hospital performing more than 19 robotic-assisted procedures annually is assumed to possess a robotic-assisted surgery system in this year

Data sources and sample characteristics differ for the two applied models: [A] a panel-data fixed effect model and [B] a random utility choice model. All hospitals that performed at least one RPE^1^ between 2006 and 2018 were included into model [A], while for model [B] all RPE patients of the largest German statutory health insurance *Allgemeine Ortskrankenkasse* (AOK) treated in 2015 and the hospitals performing their surgeries were included. RPE patients of the AOK represent around 25% of all RPE patients in Germany. Figure 1 gives an overview of the data sources, data levels and time spans used for both models.

For model [A], procedure volume, the existence of an RAS systems, hospital size (number of beds), and type of ownership were retrieved from the German (bi-)annual^2^ compulsory structured quality reports. We assume that hospitals performing ≥ 20 robotic-assisted procedures^3^ possess an RAS system and use it for most RPEs. This assumption is in line with Barbier et al. [14], who report that hospitals in possession of an RAS system tend to use it for robotic-assisted RPEs in 80–100% of the cases. Furthermore, hospitals’ certification status as prostate cancer treatment center from the German Cancer Society^4^ was added to the hospital sample.

For model [B], only data from 2015 was considered (for procedure volume data from 2014 was used due to the time lag needed, see Section Random utility choice model) and enriched with several additional variables from other sources. Similar to Kuklinski et al. [15], the data set exists of patient characteristics (8 and 10 to 12 in Fig. 1), patient travel time to the hospital of treatment (9), and hospital features (1–7). In addition to data from model [A], patient recommendation scores^5^ from the *Weiße Liste* (patient experience questionnaire data), and risk-adjusted 1-year reoperation ratios from the quality assurance with routine data program (QSR) of the AOK were added on hospital level. At the time of patient treatment observations (i.e., 2015), the 1-year reoperation ratios were not yet publicly accessible. As they constitute one of the outcome quality indicators available to research and as other indicators were also not publicly assessable, we still used the reoperation ratios for model [B].  Patients were described by their age, their Charlson Comorbidity Index value [16], the variety of choices expressed by the number of hospitals in a 30-min radius of patients’ home zip codes, and their socio-economic status (county median monthly income). Patient travel time (9) was calculated using the Stata command *orsmtime* and a local Open Source Routing Machine Server [17]. It was defined as the driving time needed with a standard passenger car under normal traffic conditions from the centroid of the patient’s home zip code area to the geographical coordinates of the treating hospital. After merging hospital data from all sources, and matching AOK patients to their respective hospital, the final data set includes 4,614 RPE patients treated in 352 hospitals in 2015.

### Panel-data fixed effect model

Model [A] analyzes the relationship between annual RPE procedure volume growth, and within hospital variation regarding RAS systems accounting for certifications, hospital size and a general, year fixed effect. We conducted a Hausman test to evaluate whether a fixed or random effect model is appropriate. Based on the Hausman test results and the initial research question, a fixed-effect (within variation) model was used. The model incorporates panel data from 2006 to 2018 from 607 hospitals. The data set was unbalanced since over the years, there was some level of fluctuation in the hospitals performing RPEs. In addition to time fixed effects, we consider hospital fixed effects. Whereas hospital fixed effects eliminate omitted variable bias from unobserved time-invariant hospital characteristics, time fixed effects remove omitted variable bias by excluding unobserved variables that evolve over time but are constant across hospitals [18]. Time-invariant heterogeneities between hospitals are reflected in the hospital-specific intercepts and time fixed effects were considered by adding year dummies into the model.

$$\mathrm{ln}{PV}_{kt}={\alpha }_{i}+ {\beta }_{rob}{Rob}_{kt}+{\beta }_{cert}{Cert}_{kt}+{\beta }_{size}{Size}_{kt}+\sum_{Y=2008}^{2018}{\delta }_{Y}{Y}_{t}+{u}_{kt}$$

where $\mathrm{ln}{PV}_{kt}$ is the natural logarithm of RPE procedure volume for year $t$ and hospital k, ${Rob}_{kt}$ and ${Cert}_{kt}$ indicate whether an RAS system was present in year $t$ or whether the hospital was a certified prostate cancer treatment center in year $t$. ${Size}_{kt}$ accounts for size changes of each hospital over time to capture a general expansion of the hospital. $\sum_{Y=2008}^{2018}{\delta }_{Y}{Y}_{t}$ captures unobserved variables that evolve over time but are constant across hospitals such as general fluctuations in overall performed RPEs in Germany.${\alpha }_{i}$ represents the hospital-specific intercept, ${u}_{kt}$ is the random error term.

While the FE model is robust against estimation biases, it cannot estimate the effect of time-invariant hospital variables [19] such as the initial level of specialization, or the type of ownership. Therefore, we divided the sample into four “volume” subsamples (< 51, 51–150, 151–300, > 300) according to their initial RPE procedure volume, and into three “ownership type” subsamples. Lastly, to enable more precise interpretation, estimation coefficients for RAS system and certification were reformulated into percentage impact, by ${e}^{{\beta }_{rob}}-1$ and ${e}^{{\beta }_{cert}}-1$.

### Random utility choice model

We are interested in whether patients prefer hospitals that use an RAS system and its effect size while accounting for other hospital characteristics such as hospital’s treatment quality, level of specialization, size, or ownership type. By using a random utility choice model [20], we estimate the utility a reference patient $i=1, \dots , I$ receives from choosing hospital $k=1, \dots , K$ at time $t=1,\dots , T$. A patient’s utility can be described by the observable utility ${V}_{ikt}$ and unobserved random utility ${v}_{ikt}$.

$${U}_{ikt} = {V}_{ikt}+ {v}_{ikt}= {{\beta }_{tt,i}TT}_{ik}+{\beta }_{{tt}^{2},i}{TT}_{ik}^{2}+{\beta }_{{tt}^{3},i}{TT}_{ik}^{3}+{\beta }_{rob,i}{Rob}_{k, t}+{\beta }_{q,i}{Q}_{k, t}^{\mathrm{^{\prime}}}+{\beta }_{c,i}{Cert}_{k, t}{+\beta }_{pv,i}{PV}_{k, t-1}+{\beta }_{z,i}{Z}_{k}^{\mathrm{^{\prime}}}+ {v}_{ikt}$$

where observable utility is estimated by travel time ${TT}_{ik}$, the existence of an RAS system ${Rob}_{k, t}$, hospital’s quality ${Q}_{k, t}^{\mathrm{^{\prime}}}$ (1-year reoperation ratio, patient recommendation score), certifications ${Cert}_{k, t}$, lagged procedure volume ${PV}_{k, t-1}$, and time-invariant hospital characteristics ${Z}_{k}^{\mathrm{^{\prime}}}$. In line with Gutacker et al. [21] and Kuklinski et al. [15], travel time is included in a non-linear form, as we expect patients’ disutility to grow quickly for shorter travel times, increase slower for medium travel times, to then increase faster for long travel times. We include 2014 procedure volume with a 1-year time lag to eliminate simultaneity bias. Demand in year $t$ cannot influence procedure volume in year $t-1$. Furthermore, by including a risk-adjusted outcome quality metric, we remove the possible simultaneity bias that might arise from a systematic discrimination of hospitals by patients, and vice versa [15, 21].

We let patients choose from a predefined choice set *M*_*it*_ of their closest 100 hospitals to their home address. We assume independent and identically distributed error terms *v*_*ikt*_. We further tested for the independence of irrelevant alternatives (IIA) assumption using the Hausman-McFadden test, and could not reject the IIA assumption [22]. Hence, we can employ a multinomial logit model (MNL) in which patient *i* chooses hospital *k* with probability *P*_*ikt*_, where *k*ʹ denotes the alternatives in the choice set of patient *i*:

$${P}_{ikt} = \frac{{e}^{{V}_{ikt}}}{{\sum }_{{k}^{\mathrm{^{\prime}}}\in {M}_{it}}{e}^{{V}_{i{k}^{\mathrm{^{\prime}}}t}}}$$

Moreover, we allow patient preferences to vary with observed patient characteristics such as age, comorbidity, number of hospitals in a 30-min radius and we express marginal utilities as:

$${\beta }_{x,i} = {\beta }_{x}+ {X}_{i}^{\mathrm{^{\prime}}}{\delta }_{x}$$

where $x$ represents each relevant independent variable. By mean-centering all variables in ${X}_{i}^{^{\prime}}$, the coefficients ${\beta }_{tt}$, ${\beta }_{{tt}^{2}}$, ${\beta }_{{tt}^{3}}$, ${\beta }_{rob}$, ${\beta }_{cert}$, ${\beta }_{pv}$, ${\beta }_{q}$, ${\beta }_{z}$ describe the preferences of a reference patient. Our results were estimated in Stata 16 using the commands *clogit* and *cmclogit.*

To enable a better interpretation of the MNL model’s coefficient estimates (marginal utilities for the reference patient), we express the coefficients in relation to travel time. This marginal rate of substitution is called willingness to travel (WTT) and allows us to infer direction and magnitude of each variable’s impact [23, 24]. Consequently, the WTT for hospitals with an RAS system compared to no RAS system can be expressed as:

$$WTT = {}^{\frac{{\partial TT}_{ik}}{{\partial Rob}_{k}}}\!\left/ \!{}_{{U}_{ik}}\right. = {}^{-\frac{{\partial U}_{ik}}{{\partial Rob}_{k}}}\!\left/ \!{}_{\frac{{\partial U}_{ik}}{{\partial TT}_{ik}}}\right.$$

$$= \frac{-{\beta }_{rob}}{{\beta }_{tt}+2{\beta }_{{tt}^{2}}TT+3{\beta }_{{tt}^{3}}{TT}^{2}}$$

The WTT for a hospitals’ prostate cancer center certification can be expressed accordingly [15]. For continuous variables such as 1-year reoperation ratio, patient recommendation score or procedure volume, the WTT is expressed in terms of standard deviation (SD) changes. We average travel time over all patients.

## Results

### Change in hospital procedure volumes – panel-data fixed effect model

**Fig. 2:**
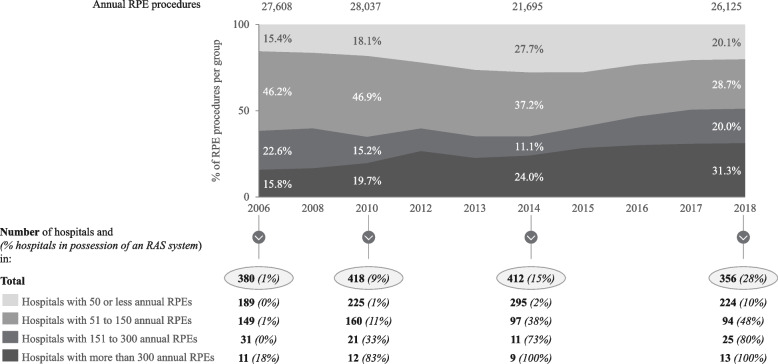
Development of procedure volume for radical prostatectomies and adoption of robotic-assisted surgery systems in Germany

The panel data of hospitals performing RPEs between 2006 and 2018 shows that total annual RPE procedure volume decreased and increased between years but stayed relatively stable over time. However, the composition of treating hospitals has changed since 2006, as can be seen in Fig. 2.

In 2006, hospitals with 50 or fewer and between 51 and 150 annual procedures together accounted for roughly 62% of total RPE procedure volume (15.4% and 46.2% respectively). This share shrank to 49% in 2018 (20.1% and 28.7% respectively). At the same time, the share of procedures being performed in very high-volume hospitals (> 300 RPEs) increased from 16 to 31%. Furthermore, the average procedure volume for very high-volume hospitals (> 300 RPEs) increased from 397 in 2006 to 628 in 2018,^6^ indicating a centralization of RPE procedures. Concurrently, the total number of hospitals performing RPEs decreased by roughly 6% from 380 in 2006 to 356 in 2018.

Furthermore, Fig. 2 shows that high-volume hospitals adopted RAS systems earlier and to a larger extent than low volume hospitals. For instance, in 2010, 83% of very high-volume hospitals possessed an RAS system while only 9% of all hospitals and a mere 1% of the hospitals performing fewer than 50 RPEs used an RAS system at that point in time. Moreover, it can be observed that between 2010 and 2018 the adoption of RAS systems reached medium-volume (51 to 150 RPEs) and high-volume (151 to 300 RPEs) hospitals: The overall share of hospitals with an RAS system for these two groups increased from 14% in 2010 to 55% in 2018 (11% to 48% and 33% to 80% respectively).

Table 1 shows the results of the fixed effect regression model for all hospitals (Model [A-1]), and for hospital subgroups by initial procedure volume (Models [A-2] through [A-5]). In total, 607 different hospitals performed at least one RPE between 2006 and 2018 and on average roughly 405 hospitals performed RPEs every year. The impact of RAS systems and certification on RPE procedure volume growth within hospitals is highly significant, except for the subgroup of very high-volume hospitals (> 300 RPEs), possibly due to its small sample size. The average effect of investing into an RAS system is an RPE procedure volume increase of 82%, meaning that between 2006 and 2018, with the introduction of an RAS system, hospitals nearly doubled the number of procedures performed prior to the use of an RAS system. This effect is even larger for initially smaller-volume hospitals increasing their procedure volume by 178% after investing in an RAS system. This difference can largely be explained by an initially lower number of performed RPEs. Still, hospitals with an initial procedure volume of 51 to 150 and 151 to 300 RPEs increased their procedure volume by roughly 58% and 59% respectively after the introduction of an RAS system. Effects for the very high-volume hospitals (> 300 RPEs) are not significant and need to be interpreted with caution. Furthermore, the impact of the RAS system is larger for private hospitals (+ 104%) than for public (+ 79%) and non-profit hospitals (+ 75%) (see Table A1 in the Appendix). Being certified as prostate cancer treatment center is also associated with growth in RPEs within all hospitals (+ 27%), and within low-volume (+ 56%) and medium-volume hospitals (+ 32%). For high and very high-volume hospitals, the impact of being certified as prostate cancer treatment center is statistically insignificant.

**Table 1:** Fixed effect (within variation) model: Impact of RAS systems and certifications on procedure volume growth

Dependent variable: ln (RPE procedure volume) – time horizon: 2006—2018										
	**Hospitals with starting**^**1**^** RPE procedure volume of:**									
	**(A-1) All hospitals**		**(A-2) 50 or lower**		**(A-3) 51–150**		**(A-4) 151–300**		**(A-5) above 300**	
*Effects*	Coeff	SE	Coeff	SE	Coeff	SE	Coeff	SE	Coeff	SE
RAS system	0.598	0.047***	1.022	0.103***	0.459	0.049***	0.462	0.068***	0.300	0.217
Prostate cancer treatment center	0.235	0.058***	0.446	0.125***	0.276	0.058***	0.046	0.075	0.338	0.405
Hospital size—number of beds	0.000	0.000	0.000	0.000	0.000	0.000	0.000	0.000	0.002	0.001*
Control variables—years										
2008	0.105	0.041*	0.296	0.068***	-0.063	0.047	-0.130	0.072	0.020	0.240
2010	-0.082	0.041*	0.151	0.068*	-0.292	0.048***	-0.313	0.074***	-0.203	0.254
2012	-0.212	0.041***	0.073	0.068	-0.506	0.049***	-0.493	0.074***	-0.001	0.276
2013	-0.440	0.042***	-0.193	0.069**	-0.695	0.049***	-0.709	0.078***	0.019	0.281
2014	-0.548	0.042***	-0.286	0.069***	-0.836	0.049***	-0.828	0.080***	0.026	0.285
2015	-0.552	0.042***	-0.273	0.070***	-0.864	0.050***	-0.856	0.080***	0.035	0.281
2016	-0.574	0.043***	-0.350	0.070***	-0.847	0.051***	-0.741	0.081***	0.066	0.281
2017	-0.594	0.043***	-0.379	0.071***	-0.870	0.051***	-0.708	0.083***	0.122	0.291
2018	-0.528	0.046***	-0.245	0.076**	-0.869	0.052***	-0.770	0.084***	0.165	0.291
Constant	3.474	0.104***	2.608	0.150***	4.270	0130***	5.506	0.240***	2.848	1.057**
Impact on RPE procedure volume^2^ of:										
RAS system	+ 82%		+ 178%		+ 58%		+ 59%		+ 35%^3^	
Prostate cancer treatment center	+ 27%		+ 56%		+ 32%		+ 5%^3^		+ 40%^3^	
Number of observations	4,048		2,138		1,496		299		115	
Number of hospitals^4^	607		373		180		35		19	
Prob > F	0.000		0.000		0.000		0.000		0.355	
R^2^ (within)	0.1618		0.1315		0.3656		0.4792		0.1380	

*Coeff* Estimation coefficient, *SE* Standard Error, 1. Hospitals are categorized by their annual procedure volume of radical prostatectomies the year they appear the first time in the database; 2. The impact of one of the variables on RPE procedure volume is transformed into percentage changes by the use of (*e*^*coef*^ - 1) * 100%; 3. Impact is not statistically significant; 4. Number of hospitals includes all hospitals having performed RPE procedure between 2006 and 2018, and can therefore deviate from the number of hospitals performing RPE procedures in a single year

^***^ *p* < 0.001

^**^ *p* < 0.01

^*^ *p* < 0.05

### Patient’s hospital choice – random utility choice model

Descriptive statistics of the 4,614 AOK patients treated in 352 hospitals (85% of all treating hospitals in 2015) for RPE in 2015 can be found in Table 2.

**Table 2:** Descriptive statistics – patient hospital choice dataset

Variables	Obs	Mean	Median (IQR)	Std. Deviation
**Radical prostatectomy**				
*Patient-level (2015)*				
Travel time [min]	4,614	34.4	25 (14–42)	31.8
Travel time past closest hospital [min]	4,614	15.6	3 (0–18)	28.7
Number of hospitals in 30 min radius	4,614	9.2	5 (2–12)	10.7
Age	4,614	66.1	66 (61–72)	7.0
Charlson comorbidity index	4,614	1.7	1 (0–2)	2.7
Median income [€]	4,614	3,100	3,164 (2,886–3,368)	428
*Hospital-level (2014)*				
Procedure volume	352	59	34 (16–61)	132
*Hospital-level (2015)*				
Robotic-assisted surgery system	352	0.19	0 (0–0)	-
Prostate cancer treatment center	352	0.12	0 (0–0)	-
Patient recommendation score	352	1.99	1.98 (1.81–2.16)	0.28
1-year reoperation ratio	352	1.22	0.74 (0.00–1.66)	1.77
Size—number of beds	352	538	441 (293–664)	364

*Obs* Observations, *IQR* Interquartile range. *Notes*: Hospital features are unweighted. Scores for patient recommendation range from 1 (best score) to 6 (worst score). We assume a hospital to possess a robotic-assisted surgery system if it performs more than 19 robotic procedures

**Fig. 3:**
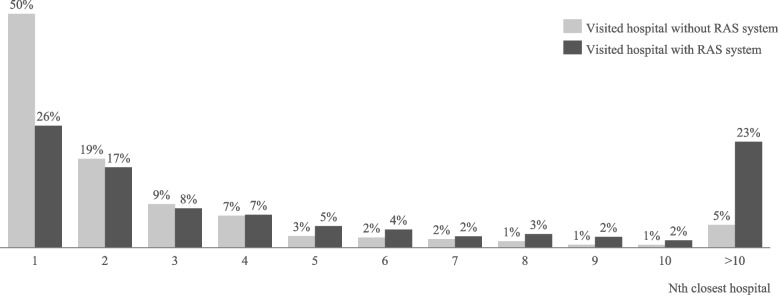
Share of patients that chose their Nth closest hospital for treatment

The average hospital had 538 beds and performed 59 RPE procedures. Of these 352 hospitals, only 19% had an RAS system and 12% were certified prostate cancer treatment centers. The average patient recommendation score was 1.99 and the average 1-year reoperation ratio was 1.22. On average, patients travelled 34.4 min to their treating hospital, passing their closest hospital by 15.6 min. The average patient was 66 years old, had 9 hospitals in a 30-min radius from home, and a comorbidity index score of 1.7. Patients treated in a hospital possessing an RAS system selected their nearest hospital in only 26% of times, compared to 50% of patients who were treated in a hospital without an RAS system (Fig. 3) – a possible reflection of the geographical availability of hospitals with an RAS system (Figure A2 in the Appendix). At the same time 23% of patients passed at least ten closer hospitals when treated in a hospital with an RAS system, compared to only 5% of patients passing at least ten closer hospitals to be treated in hospitals without an RAS system.

According to the results of the MNL model (Table 3), the reference RPE patient preferred shorter travel times, and favored hospitals that possessed an RAS system, that were certified prostate cancer treatment centers with higher annual procedure volumes, and hospitals that had better patient recommendation scores. Hospital size and type was only weakly significant for the reference patient’s utility, and variations in the 1-year reoperation ratio between hospitals did not affect the reference patient’s choice significantly. Patients older than the reference patient received more disutility from travelling longer and were influenced less strongly by the existence of an RAS system at the treating hospital relative to younger patients. Furthermore, the utility of patients with a more than average number of hospital choices in 30-min proximity was impacted more negatively by longer travel times and was more positively affected by specialization (prostate cancer treatment center, procedure volume). Patients from counties with a higher-than-average median income tended to prefer RAS less than patients from lower median income counties.

**Table 3:** Estimated marginal utilities and willingness to travel

Variable	Estimate	Standard Error	
*Main effects*			
Travel time	-0.162	0.004***	
Travel time^2^	0.001	0.000***	
Travel time^3^	0.000	0.000***	
Robotic-assisted surgery system	0.743	0.048***	
Procedure volume	0.002	0.000***	
Patient recommendation score	-0.475	0.079***	
1-year reoperation ratio	0.017	0.012	
Prostate cancer treatment center	0.466	0.048***	
Size—number of beds	0.000	0.000**	
Hospital type: non-profit vs. private	-0.168	0.065*	
Hospital type: public vs. private	-0.184	0.062**	
*Interaction with travel time*			
x Age	-0.001	0.000**	
x Comorbidity index	-0.002	0.001*	
x Number of hospitals in 30 min radius	-0.004	0.000***	
x Median income	0.000	0.000**	
*Interaction with robotic-assisted surgery system*			
x Age	-0.014	0.007*	
x Comorbidity index	0.021	0.017	
x Number of hospitals in 30 min radius	0.005	0.005	
x Median income	-0.001	0.000***	
*Interaction with procedure volume*			
x Age	0.000	0.000	
x Comorbidity index	0.000	0.000***	
x Number of hospitals in 30 min radius	0.000	0.000***	
x Median income	0.000	0.000***	
*Interaction with patient recommendation score*			
x Age	0.007	0.011	
x Comorbidity index	-0.007	0.030	
x Number of hospitals in 30 min radius	0.012	0.007	
x Median income	0.000	0.000	
*Interaction with 1-year reoperation ratio*			
x Age	-0.003	0.002	
x Comorbidity index	0.013	0.004**	
x Number of hospitals in 30 min radius	-0.004	0.001***	
x Median income	0.000	0.000**	
*Interaction with prostate cancer treatment center*			
x Age	0.012	0.007	
x Comorbidity index	-0.037	0.018*	
x Number of hospitals in 30 min radius	0.017	0.006**	
x Median income	0.000	0.000***	
			*As % of average travel time*
WTT (robotic-assisted surgery system)	7.254		*21.9%*
WTT (procedure volume)	2.849		*8.2%*
WTT (recommendation score)	1.317		*3.9%*
WTT (1-year reoperation ratio)	-0.298^1^		*-1.1%*^*1*^
WTT (prostate cancer center certification)	4.549		*13.2%*
WTT (hospital size)	0.571		*2.6%*
Number of patients	4,614		
Number of hospitals	352		
Prob > chi^2^	0.000		
Pseudo R^2^	0.573		

Multinomial logit model for colorectal resection patients treated in 2015. Coefficients represent marginal utilities. Coefficients of the interaction terms of travel time^2^ and travel time^3^, size, hospital type are not presented, but can be delivered upon request; 1. Impact is not statistically significant

^***^ *p* < 0.001

^**^ *p* < 0.01

^*^ *p* < 0.05

Translating the reference patient’s marginal utilities into WTTs, patients were willing to travel 7.3 min longer (+ 22% of average patient travel time) for hospitals that possessed an RAS system. For hospitals that were certified prostate cancer treatment centers, patients were willing to travel 4.5 min (+ 13%) longer, and for hospitals with higher procedure volume they were willing to travel 2.8 min (+ 8%) longer per SD difference. Furthermore, a one SD increase in hospitals’ patient recommendation scores, and a one SD increase in number of beds increased the WTT of the reference patient by 1.3 min (+ 4%) and 0.6 min (+ 3%) respectively. The outcome quality indicator 1-year reoperation ratio has a negligible and insignificant adverse effect on patient’s WTT.

## Discussion

The first aim of this study is to investigate the effect of RAS on treatment centralization and participating hospitals’ RPE procedure volume change between 2006 and 2018 in Germany.

Our results show that the introduction of RAS systems for RPE has considerably affected centralization of RPE treatments and the distribution of procedures among hospitals. While overall RPE procedure volume was relatively stable between 2006 and 2018, the share of RPE procedure volume performed by very high-volume hospitals increased from 16% in 2006 to 31% in 2018. We further saw in the panel-data fixed effect model that the introduction of an RAS system heavily affected procedure volume growth within all hospital groups. On average, hospitals increased their RPE procedure volume by ~ 80% after investing into an RAS system. The effect was even larger for initially low-volume (+ 178%) and private hospitals (+ 104%). Certification was an additional boost for hospitals’ procedure volume (+ 26%), especially for low- and medium-volume hospitals (+ 56% and + 32% respectively) and public hospitals (+ 28%).

The second aim of this study was to investigate whether the use of an RAS system influenced patients’ hospital choice. Our results show that the observed centralization effect is partly driven by patient preferences for RAS systems. Patients’ willingness to travel to a hospital with an RAS system was 7.3 min (+ 22%) longer than the average patient travel time of roughly 34 min. Furthermore, 23% of patients treated in hospitals that use an RAS system chose a hospital that was further away than their ten closest hospitals and almost 50% chose a hospital further away than their four or more closest hospitals indicating a deliberate, informed hospital choice: Patients take on a considerable amount of travel disutility to be treated in hospitals with an RAS system compared to hospitals not possessing an RAS system. Besides the existence of RAS systems, patients were willing to travel longer for specialization in form of higher procedure volumes (+ 8% longer than average travel time) and prostate cancer treatment centers (+ 13%). These results are in line with Kuklinski et al. [15], who investigated the effect of specialization on patients’ hospital choice for colorectal resections and knee replacement.

Lastly, our study design is independent of the assessed technology. Thus, our approach can be used to investigate similar effects of other technologies and (treatment) innovations on the hospital landscape ranging from RAS for other procedures (e.g., knee replacement) to new treatment methods such as transcatheter-aortic valve implantation and rapid recovery in orthopedics.

### Findings from other studies

Several studies have investigated the impact of the introduction of RAS systems on the centralization of RPEs. For instance, Stitzenberg et al. [25] reported a strong centralization of RPEs related to the adoption of RAS systems in New York, New Jersey and Pennsylvania from 2000 to 2009. While the number of hospitals performing RPEs decreased by 37% during that time, by 2009 the 35% of hospitals with an RAS system performed 85% of total RPE procedure volume. Similarly, Anderson et al. [26] found that the increase in RPEs in the US was driven mainly by high-volume hospitals and by those hospitals that invested in RAS systems leading to a centralization of services. Outside the USA, Riikonen et al. [27] discovered a rapid centralization of prostate cancer surgery after the introduction of RAS systems in Finland. Our results on the centralization of RPEs following the diffusion of robotic technology point in the same direction as aforementioned studies, nonetheless, the effect in Germany is not as strong.

Cheung et al. [28] showed that the increase in RAS for RPEs, and the resulting centralization are primarily driven by marketing and patient demand in the United States. Accordingly, we found that RPE patients are willing to travel considerably longer for hospitals with RAS systems, which is also in line with Stitzenberg et al. [25], who showed that average travel time of patients increased by 54% between 2000 and 2009. Furthermore, Wright et al. [29] found that hospitals in more competitive areas are more likely to invest in an RAS system to attract patients.

While hospital marketing [30] and patient perceptions [12] attribute positive procedural quality to RAS systems, the scientific evidence for a positive effect of RAS systems on outcomes and cost-effectiveness of RPE is still unclear. On the one hand, Ilic et al. [31], Yaxley et al. [32], and Wallerstedt et al. [33] reported no high-quality evidence of better oncologic outcomes between robotic-assisted RPE compared to open RPE. Short-term urinary and sexual quality of life-related outcomes as well as serious postoperative complication rates were similar [31], and also functional outcomes at 12 weeks [32] and 24 months [34] yielded equal results between robotic-assisted and open RPE. Coughlin et al. [34] concluded further that the main benefit of the robotic approach lay in its minimal invasive nature. However, in the case of Coughlin et al.’s study and also generally, a debate towards a surgeon learning curve remains and needs to be considered when analyzing effectiveness [35, 36].

On the other hand, Nyberg et al. [37] found a significant difference in erectile dysfunction in favor of robotic-assisted RPEs and Laird et al. [38] observed a positive impact of robotic-assisted procedures on outcomes such as reduced blood loss and transfusion rates. Similarly, Lindenberg et al. [39] found in a national retrospective cluster study including twelve Dutch hospitals that robotic-assisted RPE resulted in a higher chance of preservation of neurovascular bundles, better long-term urinary tract function, less blood loss, and shorter procedure times. Based on this study, Lindenberg et al. [40] recently conducted a cost-utility analysis for robot-assisted RPE from a Dutch societal perspective. The authors found an incremental cost-utility ratio in favor of robot-assisted RPE which improved even further in a best-case scenario due to economies of scale when centralizing procedures.

Overall, the question whether RAS systems can improve outcomes independent of centralization remains ambiguous – yet is highly relevant given the high investment and operating costs of RAS systems [14, 41]. Likewise, potential conflicts of interest of studies investigating the RAS impact on outcomes need to be considered, as Criss et al. [42] found that studies with financial conflict of interest appear to be associated with a higher likelihood of reporting a positive effect of RAS.

Still, centralization due to the use of RAS systems might still lead to an outcome quality increase as for RPE, like for many other procedures, higher procedure volume is linked to better outcome quality. Ploussard et al. [43], for instance, showed a correlation between hospital volume and postoperative outcomes (length of stay, complications, and hospital readmissions at 30 and 90 days) irrespective of open and minimally invasive surgery (including RPE) in a French nationwide analysis. Gershman et al. [44] found in the Nationwide Inpatient Sample of the USA that increasing volume of RPE was associated with improved perioperative outcomes up to around 100 surgeries per year. Beyond 100 surgeries, improvements appeared to be marginal.

Lastly, treatment centralization is multi-faceted: Apart from (technological) innovations, health policies such as minimum surgical caseloads or hospital capacity planning as well as changes in regional markets could drive centralization and thus limit or at least strongly influence patients’ hospital choice [45, 46]. In this context, Gutacker et al. [21] and Kuklinski et al. [15] analyze (regional) quality competition and demand changes in their studies on patients’ hospital choice. In the observed period for our study, no major health policy changes specific for RPE were introduced incentivizing treatment centralization. Still, the introduction and stepwise implementation of a flat rate payment system in Germany from 2003 to 2015 [47] could possibly have led to hospitals with low procedure volumes to discontinue care for prostate cancer. With our fixed effect model, we should be able to isolate RAS effects, however.

### Limitations

Investment in an RAS system and/or the decision to undergo the detailed, elaborate certification process to become a prostate cancer center usually echoes a broader management decision to expand the urologic department in a hospital, accompanied by for instance hiring of a new department head or additional physicians. This fact results in a potential upward bias for the coefficients of investing into an RAS system or attaining a certification. Furthermore, during the observed period, some hospitals dropped in and out of performing RPEs, creating an unbalanced data set. However, the panel data fixed effect model accounts for this problem [19].

Moreover, due to its limited cross-sectional nature, the patient choice model observed patient behavior when choosing their hospital for treatment rather than allowing for causal conclusions. To investigate a causal effect, a fixed effect random utility model with data for a longer period would be required. Additionally, our sample contains patients from one statutory health insurance potentially causing outcome biases. Still, the sample covers ~ 25% of all German RPE patients for 2015, the AOK has the largest market share in Germany (approximately 35%) and other studies have used these data as well showing their representativeness for Germany [48]. Generally, in Germany, statutory health insurances pay for the majority of health services [47], and especially for treatment of life-threatening diseases such as prostate cancer and RPE, also when performed with RAS systems. Thus, our results should not be biased by patients avoiding RAS due to extra payment.

In addition, referrals to high-volume RAS centers might explain parts of the RPE volume increases of hospitals that introduced RAS systems. These referrals could be from outpatient specialists and/or “own” referrals from a center’s urologic consultations. Unfortunately, neither for Model A nor Model B were the necessary patient level data covering the pre-operative, outpatient care path available. Future studies could add a binary variable indicating own referrals to their analyses to indicate whether RAS centers are more likely to advise surgery as treatment.

Lastly, our model potentially suffers from a simultaneity bias as hospitals treating more patients are more likely to invest into an RAS system, and the investment into an RAS system increases the number of patients treated in the respective hospital. Still, as it is likely that the number of patients (demand) influences the investment into an RAS system with a one-to-two-year time lag, it is debatable whether an actual simultaneity bias exists.

## Conclusion

Similar to other countries, there has been a rapid diffusion of RAS for RPEs in Germany leading to a centralization of treatments. This diffusion is likely driven by effective marketing and by patients’ preferences for RAS technologies. While the resulting centralization is likely to improve specialization and thus, outcome quality, there is no clear scientific evidence yet to fully support the positive outcome effect of RAS on RPEs, independent of the specialization effect. While the strategic benefit for hospital differentiation and the positive effect on centralization has become clear, encouraging RAS system adoption for RPE more broadly by health systems or payers should be considered carefully, due to the lack of clear evidence for a positive outcome effect and given the high investment and operating costs. Thus, additional research investigating the outcome effect of RAS system use is needed. Lastly, due to the high costs of RAS systems, additional research focusing on accurate RAS reimbursement should be carried out, possibly incorporating an adequate pay-for-quality scheme.

## Figures and Tables

**Fig. 4:**
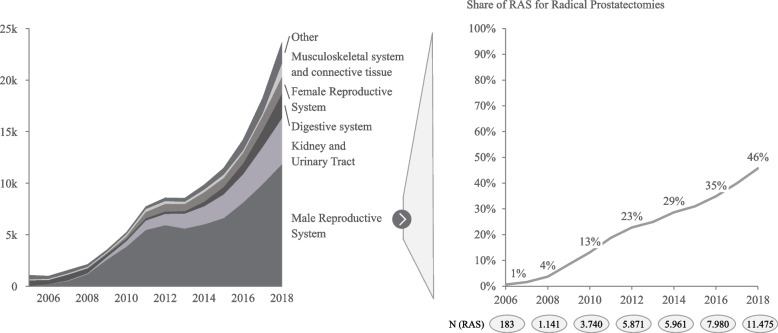
Development of robotic-assisted surgeries in Germany from 2006 to 2018. *Source:* Research Data Center of the Federal and State Statistical Offices [5]

**Fig. 5:**
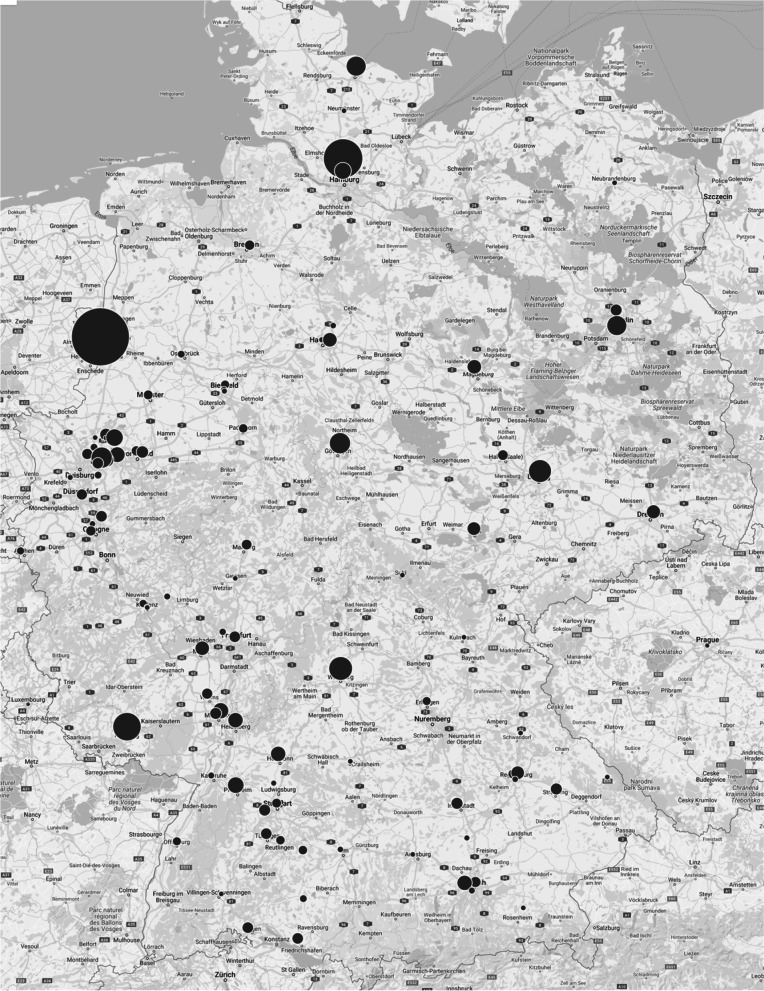
Distribution of hospitals performing robotic-assisted surgeries in Germany in 2015. *Notes*: Bubble sizes reflect the number of RPEs performed with RAS

## Data Availability

The patient-level data that support the findings of this study are available from the AOK but restrictions apply to the availability of these data, which were used under license for the current study, and so are not publicly available. Data are however available from the authors upon reasonable request and with permission of the AOK. The hospital-level data used and analyzed for this study are available from the corresponding author on reasonable request or can be requested from the Federal Joint Comission (*Gemeinsamer Bundesausschuss*, G-BA) on their website.

## Appendix

**Table:** **Table 4** Fixed effect (within variation) model: Impact of RAS systems and certifications on procedure volume growth – part two

	**Ownership type of hospitals:**							
	**(A-1) All hospitals**		**(A-6) private**		**(A-7) non-profit**		**(A-8) public**	
*Effects*	Coeff	SE	Coeff	SE	Coeff	SE	Coeff	SE
Robotic-assisted surgery system	0.598	0.047***	0.712	0.188***	0.559	0.088***	0.581	0.056***
Prostate cancer treatment center	0.235	0.058***	0.091	0.252	0.212	0.102*	0.247	0.069***
Hospital size—number of beds	0.000	0.000	0.001	0.001	0.000	0.000	0.000	0.000
Control variables—years								
2008	0.105	0.041*	0.124	0.113	0.130	0.067	0.067	0.055
2010	-0.082	0.041*	-0.022	0.112	-0.071	0.068	-0.113	0.056*
2012	-0.212	0.041***	-0.240	0.110*	-0.159	0.069*	-0.235	0.057***
2013	-0.440	0.042***	-0.598	0.111***	-0.412	0.070***	-0.385	0.058***
2014	-0.548	0.042***	-0.673	0.112***	-0.504	0.070***	-0.516	0.058***
2015	-0.552	0.042***	-0.513	0.113***	-0.551	0.071***	-0.558	0.059***
2016	-0.574	0.043***	-0.641	0.113***	-0.521	0.071***	-0.566	0.060***
2017	-0.594	0.043***	-0.779	0.113***	-0.524	0.072***	-0.550	0.061***
2018	-0.620	0.122***	-0.444	0.076***	-0.537	0.063***	-0.620	0.122***
Constant	3.474	0.104***	2.889	0.305***	3.510	0.142***	3.676	0.159***
Impact on RPE procedure volume of:								
Robotic-assisted surgery system	+ 82%		+ 104%		+ 75%		+ 79%	
Prostate cancer treatment center	+ 27%		+ 10%^2^		+ 24%^2^		+ 28%	
Number of observations	4,048		754		1,441		1,853	
Number of hospitals^3^	607		119		244		272	
Prob > F	0.000		0.000		0.000		0.000	
R^2^ (within)	0.1618		0.2098		0.1509		0.1558	

*Coeff* Estimation coefficient, *SE* Standard Error, 1. Hospitals are categorized by their annual procedure volume of radical prostatectomies the year they appear the first time in the database; 2. Impact is not statistically significant; 3. Number of hospitals includes all hospitals having performed RPE procedure between 2006 and 2018, and can therefore deviate from the number of hospitals performing RPE procedures in a single year

^***^ *p* < 0.001

^**^ *p* < 0.01

^*^ *p* < 0.05

## Abbreviations

AOK: Allgemeine Ortskrankenkasse
IIA: Independence of Irrelevant Alternatives
MNL: Multinominal Logit Model
RAS: Robotic-Assisted Surgery
RPE: Radial Prostatectomy
SD: Standard Deviation
WTT: Willingness To Travel

## Footnotes

1 We identify RPEs with the procedure code 5–604 from the German Operation and Procedure Catalogue (*Operationen- und Prozedurenschlüssel*, OPS).

2 From 2006 to 2012, structured quality reports were published bi-annually, from 2012 onward annually.

3 We identify robotic-assisted procedures with the OPS procedure code 5–987.

4 For the full list of centers see https://oncomap.de/centers

5 Recommendation is addressed by question no. 14: “Would you recommend this hospital to your best friend?”.

6 The average procedure volume per hospital of a hospital sub-group can be calculated by multiplying the sub-group’s procedure volume share with the total procedure volume and dividing the result by the number of hospitals in the respective hospital sub-group.
